# Novel Covalently Linked Insulin Dimer Engineered to Investigate the Function of Insulin Dimerization

**DOI:** 10.1371/journal.pone.0030882

**Published:** 2012-02-17

**Authors:** Tine N. Vinther, Mathias Norrman, Holger M. Strauss, Kasper Huus, Morten Schlein, Thomas Å. Pedersen, Thomas Kjeldsen, Knud J. Jensen, František Hubálek

**Affiliations:** 1 Diabetes Research Unit, Novo Nordisk A/S, Novo Nordisk Park, Måløv, Denmark; 2 Faculty of Life Sciences, IGM, University of Copenhagen, Frederiksberg, Denmark; University of South Florida College of Medicine, United States of America

## Abstract

An ingenious system evolved to facilitate insulin binding to the insulin receptor as a monomer and at the same time ensure sufficient stability of insulin during storage. Insulin dimer is the cornerstone of this system. Insulin dimer is relatively weak, which ensures dissociation into monomers in the circulation, and it is stabilized by hexamer formation in the presence of zinc ions during storage in the pancreatic β-cell. Due to the transient nature of insulin dimer, direct investigation of this important form is inherently difficult. To address the relationship between insulin oligomerization and insulin stability and function, we engineered a covalently linked insulin dimer in which two monomers were linked by a disulfide bond. The structure of this covalent dimer was identical to the self-association dimer of human insulin. Importantly, this covalent dimer was capable of further oligomerization to form the structural equivalent of the classical hexamer. The covalently linked dimer neither bound to the insulin receptor, nor induced a metabolic response *in vitro*. However, it was extremely thermodynamically stable and did not form amyloid fibrils when subjected to mechanical stress, underlining the importance of oligomerization for insulin stability.

## Introduction

Insulin, a small peptide hormone, is crucial in maintaining blood glucose homeostasis. Defects in insulin secretion and action result in diabetes mellitus, a severe metabolic disorder, which untreated will lead to serious health problems and ultimately death [1]. Insulin consists of 51 amino acids in two peptide chains, the A- and the B-chain. It contains six cysteine residues (Cys) forming three disulfide bonds, two of them link the two chains and one intra-chain bond is found in the A-chain [2]. Insulin is expressed in the pancreatic β-cells of the islets of Langerhans [3]. At micromolar concentrations it self-associates into dimers. This concentration is found during expression in the β-cells and there are indications that dimers are formed in the ER during expression [4], [5]. The positions present in the dimer forming surface are all located in the B-chain and involve residues: B8, B9, B12, B13, B16, and B23–B28 [2], [6]. At millimolar concentration in the presences of zinc ions, insulin further associates into hexamers [7]. When blood glucose levels are low, insulin is stored as hexamers in vacuoles of the β-cells. In response to elevation of blood glucose levels, the hexamers are released into the blood where they dissociate into dimers and monomers [8]. Insulin mediates its effect through the insulin receptor [9]. The insulin receptor is found as a homo-dimer and it is believed that two separate monomers bind to the receptor. It is believed that the function of dimer and hexamer formation lies in stabilisation of the molecule during storage [10], [11].

Dimerization using disulfide bonds is a common strategy for protein stabilisation often found in nature [12]–[14]. Introduction of Cys in the dimer forming surface of a protein can lead to the formation of disulfide-bridged dimers [15]–[21]. Crystal structures (ex. PDB code 1mso) of human insulin dimers reveals that residues in positions B12, B13, B24, B25 and B26 in one monomer are found in close proximity to their complementary position in the second monomer (Cα to Cα distance <10 Å) (Figure S1) but only the distance between the two B25 residues is below 6.5 Å which is ideal for disulfide bond formation [22]. We have previously observed that introduction of a Cys in position B25 results in exclusive expression of a covalent dimer insulin precursor (see experimental section) whereas substitution of other positions in the dimer surface with Cys led to negligible or no expression of insulin precursors [23]. The B25C position is therefore a unique position in insulin with respect to dimer formation.

In this article, we report structural and functional properties of this B25C covalently linked dimer (B25C-dimer) in comparison to human insulin and discuss the balance between oligomerization and receptor binding.

## Methods

### Plasmids construction and expression

Material, vector, strain and construction were as previously described [24]–[27]. Briefly, the mutation to a Cys was introduced in the insulin coding sequence by overlapping PCRs [28] in the selected position. The insulin precursor is expressed in *Saccharomyces cerevisiae* and secreted as a proinsulin-like single-chain consisting of a spacer Glu-Glu-Ala-Glu-Ala-Glu-Ala-Pro-Lys (EEAEAEAPK) [25] followed by the B-chain (B1–B29) linked to the A-chain (A1–A21) by a mini C-peptide Ala-Ala-Lys (AAK) [29]. The expression yield of the insulin precursor were determined by reversed-phase high-performance liquid chromatography (RP-HPLC) based on peak area using human insulin as external standard. The was mass determined by liquid chromatography/mass spectrometry (LC/MS) as previous described [23].

### Purification

A 3 L batch of the B25C precursor was fermented in shaking flasks. The cell-free culture supernatant was acidified and the precursor was partially purified and concentrated by a capture step using ion exchange chromatography. The precursor was enzymatically digested to remove the spacer and C-peptide, the digest was stopped after several days and the conversion verified by LC/MS. The dimer was further purified by RP-HPLC and lyophilized.

### Construction of the B25C alkylated monomer

Partially purified dimer using ion exchange chromatography was concentrated 10 times by lyophilisation and subsequent dissolution in water to a concentration of 1.5 mg/ml. Tris(2-carboxyethyl)phosphine (TCEP) immobilized on agarose gel (Thermo Scientific) was equilibrated in elution buffer (from cation exchange chromatography) mixed 1∶1 with 1 M NaOAc (sodium acetate) pH 5.5. The liquid was removed from 3 ml TCEP slurry and the concentrated solution with the dimer precursor was added. The sample was incubated over night with slow mixing, just fast enough to ensure gel suspension. 100 µl 10 mM N-ethyl maleimide (NEM) in water was added and set to react for 1 hour with turning. The samples were separated from the TCEP gel by centrifugation and the two B25C-NEM monomeric isomers were purified by RP-HPLC. The fractions for each isomer were pooled and concentrated using a speed vac.

### Pulse-chase

The experiment was carried out essentially as described before [25]. Briefly, cultures with OD_600_ of approximately 12 in media without Cys and Met were pulsed with 30 µCi [^35^S]cysteine(PerkinElmer) for 2.5 min followed by removal of the labelled Cys. Media containing 18.5 mM un-labelled Cys and Met were added equal to 5.5 min after the start of pulsing and the sample was taken. Immediately afterwards, protein synthesis was stopped using 1 µl 5% sodium azide. The cells were spun down and separated from the supernatant. The samples were then frozen at −20°C until further analyses.

Analyses of the samples were performed by SDS-PAGE. The cells were lysed and the cell lysate and the supernatant was applied to a SDS-gel (10% Bis-Tris, Invitrogen) either untreated or following treatment with PNGase F (Biolabs) for 30 min at 37°C. The gels were fixed, dried and then exposed to a phosphor screen and scanned in a phosphor imager (Typhoon 9410, Variable Mode Imager, GE Healthcare).

### Crystal structure

Crystals were grown by the sitting drop vapour diffusion method. 1 µl of protein solution containing 4.6 mg/ml of the insulin analogue was mixed with 1 µl of reservoir solution containing 0.1 M Bis- Tris pH 6.5 and 3.0 M NaCl. Crystals with dimensions of about 0.1 mm were obtained within 1 week. The crystal used for data collection was soaked in 15% ethylene glycol prior to freezing in liquid nitrogen. Data were collected in house using a rotating anode (Rigaku MicroMax-007HF, Cu/Kα radiation, λ = 1.5418 Å) and a Rayonix SX-165 CCD detector (Mar Research, Hamburg) at a temperature of 100 K. The data were indexed and scaled with the HKL2000 package. The structure was solved by molecular replacement method using MOLREP [30] with an in house insulin monomer as search model. The structure was refined in Refmac [31], atom coordinates were manually adjusted in Coot [32]. Water molecules were added using the ‘Find water’ algorithm in Coot. Data collection details and refinement statistics are summarized in Table 1. Coordinates with structure factors have been deposited to the Protein Data Bank (PDB) with the accession code 3U4N.PDB

**Table 1:** Data collection and refinement statistics.

*Data processing statistics*	
wavelength (Å)	1.5418
Space group	I2_1_3
cell axis a, b, c (Å)	77.9
cell angles (deg) α β γ	90
temperature (K)	100
diffraction limit (Å)	1.98
no. of observations	38677
unique reflections	5631
highest resolution shell (Å)	2.01-1.98
completeness	
all data (%)	99.3
high. resolut. shell (%)	85
R_merge_ a	
all data (%)	6.7
high. resolut. shell (%)	25.8
*Refinement statistics*	
resolution range (Å)	31.8-1.98
no. of reflections	5334
R value (%) b	16.7
R_free_ value (%)^b^	21.7
highest resolution shell (Å)	2.03-1.98
no. of reflections	316
completeness (%)	89.8
R value (%)^b^	18.0
R_free_ value (%)^b^	29.4
r.m.s.d.^c^	
bond length (Å)	0.022
bond angles (deg)	2.417
average B-factor (Å^2^)	23.1

a *R_merge_ = Σ|I_i_−I|/ΣI where I_i_ is an individual intensity measurement and I is the mean intensity for this reflection.*

b *R value = crystallographic R-factor = Σ|F_obs_|−|F_calc_|/Σ|F_obs_|, where Fobs and Fcalc are the observed and calculated structure factors respectively. R_free_ value is the same as R value but calculated on 5% of the data not included in the refinement.*

c *Root-mean-square deviations of the parameters from their ideal values.*

### Analytical Ultracentrifugation

Analytical Ultracentrifugation (AUC) experiments were performed with a BeckmanCoulter XL-I analytical ultracentrifuge (Indianapolis, IN), using the interference optics of the instrument. Samples were filled in sapphire-capped two-sector Epon-centrepieces of 12 mm optical pathlength. The B25C-dimer was dissolved in and dialysed against buffer containing 50 mM sodium chloride, 10 mM Tris at pH 7.4. The dialysate was used for dilution and optical referencing. All experiments were conducted at 20°C and with multiple concentrations of the peptide, prepared by 7-fold serial dilution. Sedimentation Velocity (SV) experiments were performed at 48 krpm and analysed with either the c(s)-algorithm [33] as implemented in SEDFIT v11.8 or by the time-derivative method [34], as implemented in DCDT+ v2.3.1. Sedimentation Equilibrium (SE) experiments were performed at sequentially 15, 24 and 36 krpm and attainment of apparent hydrodynamic and thermodynamic equilibrium was ascertained with MATCH. Water blanks for each cell and speed were recorded immediately after the experiment and subtracted from the raw data before analysis. SE data were globally fitted to multiple models of reversible self-association with NONLIN [35] and the best-fit model selected based on a minimised variance and visual inspection of the residuals run pattern. SV experiments of the B25C-dimer in the presence of Zn^2+^ were performed at a constant concentration of peptide of 75 µM and different concentrations of Zn^2+^. The ratio of [Zn^2+^]/[B25C-dimer] was adjusted to the concentration of insulin monomer normals, i.e. one mole of B25C-dimer is two normal with respect to insulin monomers. The partial specific volume 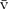 of the B25C-dimer and the density ρ of the buffer were measured with a digital densitometer DMA5000M from AntonPaar (Graz, Austria), the viscosity η of the buffer was calculated from composition using SEDNTERP.

### Receptor binding assay

Receptor binding was measured by an IR competition binding assay performed on the A isoform of the insulin receptor in a scintillation proximity assay (SPA) as previously described [24]. Briefly, binding competition of the B25C-dimer and [^125^I]TyrA14-labelled insulin (Novo Nordisk A/S) in the SPA assay was used to determine binding receptor affinities. A human standard (n = 4) and the dimer (n = 4) were tested in one plate. The data was analysed according to a four-parameter logistic model [36] and the affinities were expressed relative to a human insulin standard [IC_50(insulin)_/IC_50(analogue)_×100%].

### Metabolic potency determination

The metabolic potency was determined by lipogenesis essentially as described before [37], [38]. Shortly, isolated primary rat adipocytes were shaken vigorously for 1 h at 37°C. Aliquots of 100 µL were distributed in 96-well PicoPlates and incubated 2 h at 37°C with gentle shaking together with 10 µL glucose solution containing D-[3-^3^H]glucose and glucose and 10 µL of increasing concentration of HI (for reference) or B25C-dimer. The incubation was stopped with addition of 100 µL MicroScint E (Packard) and the plates were counted in a TopCount NXT (PerkinElmer Life Science). The data were analyzed according to a four-parameter logistic model [36] and the metabolic potency was expressed relative to a HI standard [EC_50(insulin)_/EC_50(analogue)_×100%].

### DSC

The differential scanning calorimetry (DSC) measurements were performed essentially as described before [11]. The insulin analogues were formulated at 0.2 mM in a 2 mM phosphate buffer at pH 7.5 which was also used as reference buffer. The samples were heated from 10°C to 110°C with a scan rate of 60°C per hour.

### ThT fibrillation assay

Samples were prepared freshly before each assay. Thioflavin T (ThT) (Sigma) was added to each sample from a concentrated stock solution in water to a final concentration of 1 µM. Four aliquots of 200 µl from each sample were placed in a 96 well microtiter plate (Packard OptiPlate™-96, white polystyrene). The plate was sealed with Scotch Pad (Qiagen). The assay was performed using a Fluoroskan Ascent FL fluorescence plate reader (Thermo). The temperature was adjusted to 37°C and the orbital shaking was adjusted to 960 rpm (1 mm amplitude). Fluorescence measurements were done using 444 nm and 485 nm excitation and emission filters, respectively. The plate was incubated for 10 min without shaking prior to the first measurement and then measured every 20 minutes for 45 hours. Between each measurement, the plate was continuously shaken and incubated as described. The background fluorescence emission from ThT in the absence of amyloid fibrils was negligible and thus no background correction was done. Each shown time point is the mean of the four replicas with standard deviation error bars. Only data obtained in the same experiment (i.e. samples on the same plate) are presented.

Insulin concentrations were determined by RP-HPLC methods using an insulin standard as a reference. After completion of the ThT fibrillation assay pools were made by recollecting 150 µl from each replica. These pools were centrifuged at 30,000 g for 40 minutes. The supernatant was filtered through a 0.22 µm filter and the concentration of insulin remaining in solution determined by RP-HPLC analysis.

## Results

### Expression of the B25C-dimer

RP-HPLC analyses of HI and the B25C-dimer fermented under the same conditions showed that the B25C-dimer had an expression yield of 108% (n = 3, SD = 1.98%) relative to that of HI (based on UV absorbance of HI). LC/MS analyses of the fermentations showed that the B25C precursor was present solely as a dimer, which was observed only for this precursor in the Cys scan of insulin [23]. A small second peak (∼4% of total) with equal mass to the B25C- dimer precursor was also observed and may represent another conformation either in form of disulfide scrambling, alternative folding, etc. (Figure S2).

Pulse-chase experiments were used to investigate the point of dimer formation. Disulfide bonds are readily formed in the oxidizing environment found in the cellular compartments of the secretory pathway [39]. Also, during expression in yeast the concentration is high enough for dimer formation [40]. Thus, if the insulin self-associates to dimers bringing the two B25 Cys in proximity to each other this allows for disulfide bond formation. The pulse-chase experiments showed the presence of the covalent dimer in cells demonstrating that the formation occurs during expression (Figure S3). This gives strong indications that HI forms dimers during expression in yeast as proposed by Kjeldsen [40].

### Purification of the dimer

The B25C-dimer precursor was fermented, partially purified and up-concentrated using ion exchange chromatography. Insulin precursors are quickly converted into mature insulin analogues by removal of the spacer and C-peptide by enzymatic digestion. However, as the C-peptide was only slowly removed from the B25C-dimer, it was necessary to digest for several days. Under these harsh conditions large amounts of a by-product were also generated (Figure S4). The B25C-dimer was further purified by RP-HPLC and lyophilized.

### The covalently linked dimer versus non-covalent human insulin dimer

**Figure 1:**
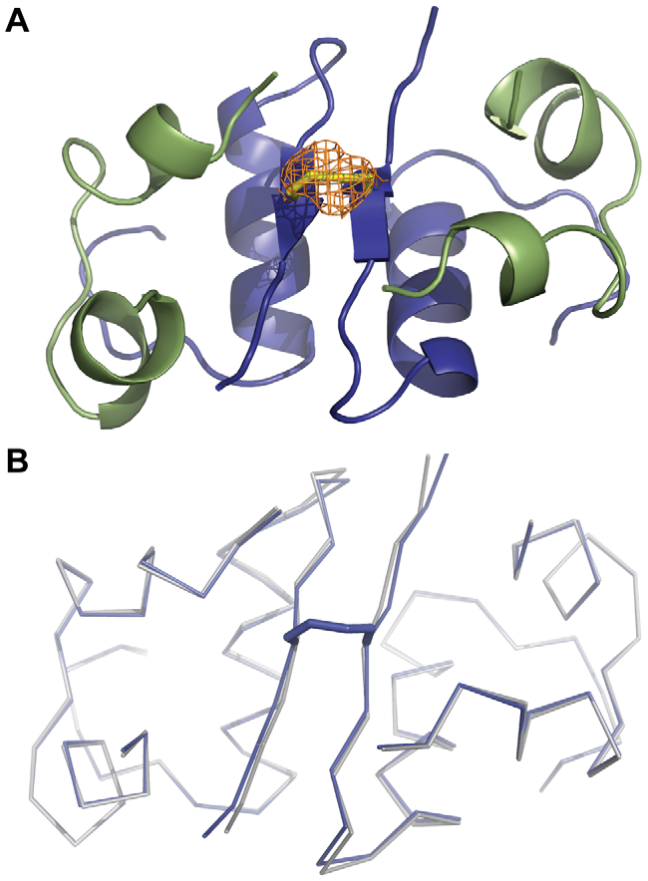
Cartoon representation of the crystal structure of the B25C-dimer. **A:** The A chain is coloured in green and the B chain is shown in blue. The additional disulphide bond is shown by stick representation (yellow). An omit map was calculated by omitting the Sulphur atom of B25C. The resulting difference electron density Fo-Fc map is coloured in orange at σ-level = 3.0. It is clear from the structure that the two monomers are linked by a disulfide bond between the two adjoining B25C. **B:** Comparison of the B25C structure (blue) with that of the porcine in-sulin (PDB code 1B2E) (grey). The Cα trace shows that the two structures have a high resemblance with minor deviations in Cα positions at residue B21E and B29K.

The structure of the B25C-dimer was determined by X-ray crystallography and refined to a resolution of 2.0 Å. The crystals belonged to the cubic space group I_213_ with cell dimensions a = b = c = 77.9 Å with one monomer in the asymmetric unit. The unit cell axes were ∼1 Å shorter compared to a typical cubic insulin crystal [41], [42]. The dimeric structure was formed by applying appropriate symmetry operations. The crystal structure (Figure 1) reveals that disulfide bonds were formed correctly with no disulfide scrambling and the two monomers were linked via a new disulfide bond formed between the introduced Cys in position B25 of each monomer. The crystal structure of the B25C-dimer resembled that of porcine insulin (B30T→B30A compared to HI, PDB code 1B2E) crystallized in the same space group). A structural alignment of the two structures yields an RMSD of 0.20 Å (all Cα) with the largest deviation found at position B21E. The backbone of the B-chain C-terminus is slightly shifted relative to the 1B2E structure. This is most likely caused by a small difference of the B21E position and its effect on the crystal packing interaction with the C-terminal part of the B chain in a symmetry related molecule. The residues close to the additional disulfide bond (B23–B28) had an RMSD value of 0.39 Å (Cα). The distance, between adjacent B25 positions in the two monomers forming a dimer, increased by 0.3 Å in the B25C-dimer. Overall, no significant conformational perturbations caused by the introduction of the additional disulfide bond at position B25 could be observed.

#### Self-association of the B25C-dimer

**Figure 2:**
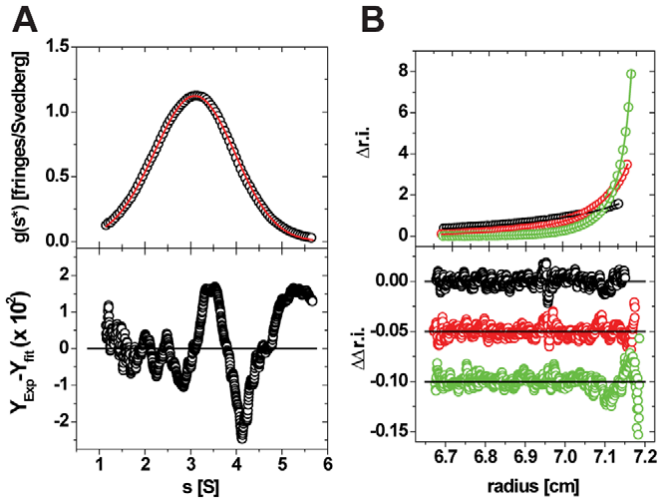
AUC results for the B25C-dimer. **A:** SV Analysis of the B25C-dimer in the presence of 2 Zn^2+^/hexamer (insulin normals). In the top part of the figure, open circles represent the g(s*)/s-curve derived from a dcdt-analysis. For clarity, only every 10^th^ data point is shown. The solid red line represents the fit to a model of a single ideal species, resulting in the parameters shown in Tabel 2. The bottom part of the figure represents the local deviations between the experimental and simulated data (residuals). Every data point is shown. The rmsd of the shown fit is 9.83×10^−3^. **B:** Representative data of a SE experiment used to determine the self-association model of B25C. In the top part of the figure, open circles represent experimental concentration distributions at apparent thermo- and hydrodynamic equilibrium for one concentration (out of five) at 15 krpm (black), 24 krpm (red) and 36 krpm (green). For clarity, only every 10^th^ data point is shown. The solid like-colored lines represent the global fit to all measured conditions to a model of a reversible monomer-dimer model, resulting in the equilibrium coefficient mentioned in the text. The bottom part of the figure represents the local deviations between the experimental and simulated data (residuals). Every data point is shown. The molar mass parameter was fixed to its expected value and the global rmsd of the fit is 7.4×10^−3^.

AUC was used to investigate the B25C-dimer self-associating abilities and compared to those of known oligorimization of HI. SV experiments showed that the B25C-dimer had a typical pattern for a reversible self-association, qualitatively similar to HI, (Figure S5,A). SV measured at constant peptide concentration and increasing [Zn^2+^]/[B25C-dimer] from 0 to 6 showed formation of larger oligomers. At [Zn^2+^]/[B25C-dimer] ratios of 2 and 3, the oligomers in solution were almost exclusively trimers of the B25C-dimer (analogous to hexamers of monomeric insulin, Figure 2,A). This was indicated by an average molar mass of the macromolecular compound close to that expected for a trimer and values for the diffusion and sedimentation coefficients are in agreement with published values for hexameric insulins of various origins, (Table 2). Increasing the [Zn^2+^]/[B25C-dimer] ratios resulted in even higher oligomeric species. This behaviour again resembles that of HI (Figure S5,B), confirming the high resemblance of the B25C-dimer to HI in respect to its self-association ability.

**Table 2:** The experimental parameters determined from the fit in Figure 2 and results previously determined for hexameric insulin of human and porcine origin.

Sample	Standard Sedimentation Coefficient s_20,w_, [S]	Standard Diffusion Coefficient D_20,w_, (×10−7 [cm^2^/s])	Molar mass [kg/mole]	R_h_ [nm]
B25C-dimer	3.24*	9.01*	35.56*	2.38
HI [65] **	3.02*	7.93	34.85	2.75
Bovine insulin [66]	3.12	8.35*	34.4	2.63
Porcine insulin [67]	3.09	8.17*	34.67	2.7

* *Measured value.,*

** *Probably affected by non-ideality because of high concentration.*

The equilibrium coefficient for the dimer formation of B25C-dimers was measured by sedimentation equilibrium experiments and the results indicated monomer-dimer equilibrium with K_D1–2_ of 1.32×10^−4^ [M], (Figure 2,B). This coefficient for the formation of dimers of the B25C-dimer (corresponding to insulin tetramers) is of the same order of magnitude as observed for formation of both the classic dimer surface and dimer formation through the hexamer surface when calculated based on an indefinite duoisodesmic association model (indefinite monomer on monomer association) of insulin [43], [44].

#### *In vitro* activity

**Figure 3:**
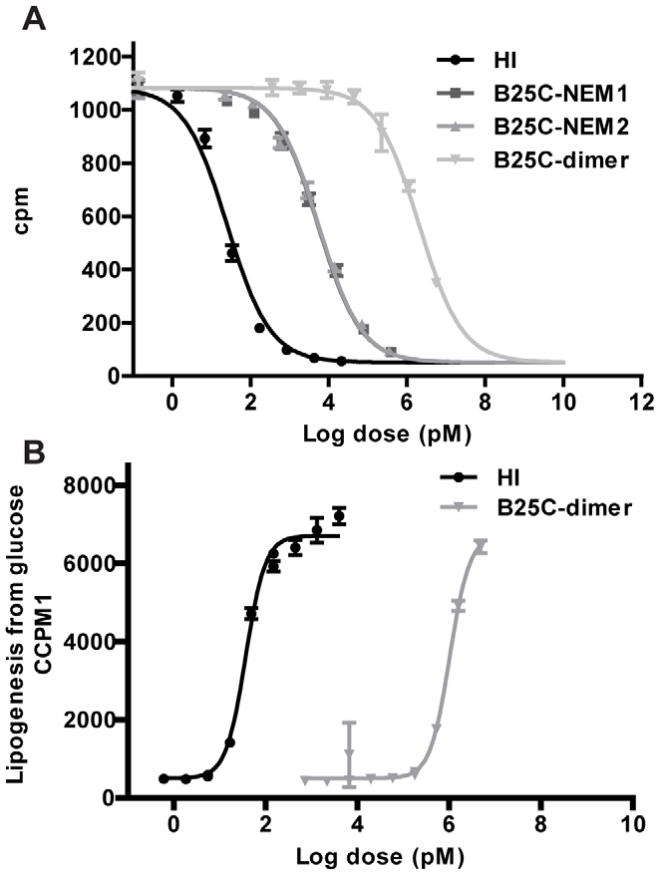
Measurements of *in vitro* activity of the B25C-dimer compared to HI. **A:** Representative insulin receptor binding curves for HI(black), B25C-NEM1 (dark gray) B25C-NEM2(gray)and the B25C dimer(light gray). **B:** Representative metabolic dose response curves for HI(black) and the B25C-dimer (dark gray). Each point on the graph represents the mean ± SD, n = 4 within one assay.

The B25C-dimer was investigated with regard to its binding affinity to the receptor in a receptor binding competition assay. The dimer was found to bind with markedly decreased affinity compared to HI (IC50 = 0.0012% relative to HI in the same plate, n = 3, SD = 0.00008%), (Figure 3A). The B25 position is important for receptor binding [45], [46] and to investigate if the decrease in binding affinity was caused by the substitution of Phe in position B25 with Cys a monomeric form of the analogue was constructed. The B25C-dimer precursor was reduced using immobilized TCEP and subsequently alkylated with NEM. The alkylation step was necessary to protect the free thiol on the B25 Cys from disulfide scrambling during the assay, which was performed at physiological pH. The introduction of the NEM moiety resulted in two stereoisomers of B25C-NEM [23], which gave rise to two isolated peaks in the LC analysis. Both forms of the monomer were purified and tested separately in the receptor assay. There was no difference in the binding affinity of the two isomers (B25C-NEM1:IC50 = 0.35%, SD = 0.015% and B25C-NEM2:IC50 = 0.34%, SD = 0.029% relative to HI in the same plate, n = 3), (Figure 3A). The binding of the B25C-monomer was therefore more than 250 times stronger than that of the B25C-dimer. We thus concluded that the mutation of the Phe to a Cys was not the main cause of the low affinity of the dimer.

The ability of the B25C-dimers to elicit a metabolic response was tested in a lipogenesis assay. The results reflect those seen in the receptor assay with EC50 values 0.003% (relative to HI in the same plate, n = 3, SD = 0.0003%), (Figure 3B). This indicates that the low binding measured in the B25C-dimer assay was not unspecific as an *in vitro* response was seen in the same range as the binding affinity.

#### Stability

**Figure 4:**
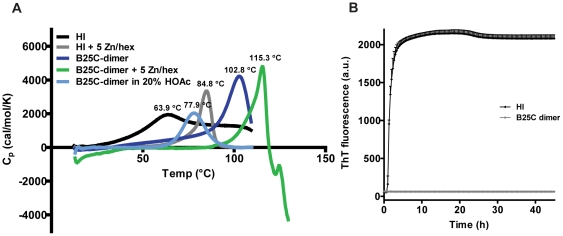
Assessing the stability of the B25C-dimer compared to HI. **A:** DSC of HI and the B25C-dimer. **B:** ThT fibrillation assay of 0.3 mM B25C-dimer (grey diamonds) and 0.6 mM HI (black diamonds) with incubation at 37°C and vigorous shaking as described in “Methods”. Both samples contained 7 mM phosphate adjusted to pH 7.4.

The thermodynamic stability of the B25C-dimer compared to HI was investigated by DSC. The excess heat capacity (Cp) of the samples compared to a reference buffer is shown in the thermograms, (Figure 4A). The transition midpoint (taken as the maximum of the thermograms) of the melting curve (T_m_) for the B25C-dimer (T_m_ = 102.8°C) was increased by 38.9°C when compared to HI (T_m_ = 63.9°C). Huus et al. [11] have previously shown with a combination of DSC and circular dichroism that thermal denaturation of the monomer proceeds through a non-two-state transition with an intermediate, whereas the insulin hexamer formed in the presence of zinc ions (Zn^2+^) proceeds through a two-state transition where the denaturation of the monomer occurs instantaneously following dissociation of the hexamer. Addition of zinc ions to HI resulted in hexamer formation which caused an increase in the T_m_ of HI to 84.8°C as demonstrated before [11]. When zinc ions were added to the B25C-dimer an increase was also observed to even higher extend with a T_m_ well above 100°C. Above the transition temperature rapid aggregation and precipitation was indicated by a steep exotherm. Denaturation of the dimer was not reversible and it was not possible to calculate thermodynamic parameters. Acetic acid (HOAc) has been shown to promote dissociation so that insulin is found predominantly in its monomeric form [47]. The B25C-dimer dissolved in 20% HOAc displayed an endothermic peak with a subsequent baseline making it possible to calculate thermodynamic parameters. The thermogram for the B25C-dimer in 20% HOAc fitted well to a non-two-state transition model which indicates that intermediary states are present. The calorimetric enthalpies (ΔH) were obtained for the dimer in 20% HAc and HI at pH 7.4 with and without zinc. The stability of the B25C-dimer (132.4 kJ/mol; mol refers insulin normals) was markedly increased compared to HI (87.8 kJ/mol). With the addition of zinc ions to HI the enthalpy increases to 145.4 kJ/mol which is only slightly more than the B25C-dimer at acetic pH. The T_m_ of the dimer in 20% HOAc had decreased to 77.9°C compared to the neutral pH. It is unclear whether the acidic pH only influences the oligomerization or whether it affects the thermodynamic stability of the B25C dimer molecule as well. It has been shown that insulin in 20% HOAc retains a native like structure [47], but it is likely that the low pH causes changes of charge states resulting in a decrease in stability.

Even though insulin is a relatively stable protein because of its tight conformation caused by the three disulfide bonds [2], it still has flexible areas mainly in the C- and N-terminals of the B-chain. One of the largest physical stability issues with insulin is fibrillation which is thought to be initiated by unfolding of the C-terminal end of the B-chain [48]. The propensity of the B25C-dimer to form amyloid fibrils was compared to HI in a ThT fibrillation assay, (Figure 4B). HI fibrillated fast within the first hour of incubation at 37°C with vigorous shaking, but remarkably no fibrillation of the dimer was observed within 45 hours. Furthermore, after completion of the ThT fibrillation assay the concentration of B25C-dimer in solution was unchanged whereas all HI was lost from solution due to amyloid fibril formation (data not shown).

## Discussion

In the crystal structure of HI the two B25 positions are located in the C-terminal β-sheets of the respective B-chains just opposite each other with a distance suitable for disulfide bond formation (Cα to Cα distance <6.5 Å) [22]. Introduction of a Cys in position B25 resulted in expression of a dimeric precursor. A disulfide bond linking the two Cys, one from each insulin molecule, was clearly visible in the electron density map from the crystal structure analysis. AUC analyses showed that the B25C-dimer oligomerization pattern resembled that of HI and that it was capable of forming analogous hexamers in the presence of zinc ions.

Insulin has a complex oligomerization pattern which stabilizes the protein during storage. Other proteins favour covalent dimerization through disulfide formation [12]–[14]. To assess the reason for the highly evolved oligomerization pattern of insulin, the B25C-dimer was used to investigate the effect of the first oligomeric state on insulin's activity and stability.

The expression yield of an analogue reflects its stability and the yeast cells ability to fold it [40], [49]. Chaperones are known to play an important role in nascent protein stabilization and folding [5]. Similar to chaperones, insulin oligomerization during expression could play an important role for the ability of the yeast cells to export it into the supernatant [40]. It could be argued that dimerization also causes higher stabilisation during expression compared to the monomeric form. It would therefore be expected that the expression yield of the B25C-dimer be higher than that of HI if this was present as a monomer during expression. The fact that the expression yields of both insulin analogues are similar indicates that HI is present as a dimer during expression in yeast. Until now it has been speculated that insulin associates into dimers in the ER, as it was estimated that the concentration is high enough to allow insulin dimer formation [40]. The requirement for proper folding of the self-associated dimer prior to B25C disulfide bond formation is inferred from the results of insulin's cysteine scan. Although cysteine substitutions in 60% of insulin's positions resulted in expression in yeast, only B25C substitution led solely to dimer formation. In fact, the pulse-chase experiment reported here for the B25C-dimer demonstrated that insulin is indeed associated into dimers during expression and this association likely occurs early in the secretory pathway.

It is believed that it is the monomeric form of insulin that binds to the insulin receptor even though the receptor forms a dimer and therefore in principle could bind the HI-dimer [9]. In spite of large efforts the structure of the complex between insulin and its receptor is as of yet unavailable and many aspects of insulin binding to its receptor are still unclear. Still much information about the binding is available from other experiments [2], [24], [27], [45], [50]. It is well known that B25F is important for binding of insulin to its receptor [46], [51] and replacement of Phe with a Cys could be a factor in loss of binding. The receptor binding affinity had decreased from picomolar affinity observed for HI to nanomolar affinity for B25C-NEM confirming the importance of the B25 position for receptor binding. Remarkably, affinity for B25C-NEM was still 250 times higher than that of the B25C-dimer. One possible explanation for the binding observed for the B25C-dimer is, that small amounts of impurities (below detection limits) resembling the monomeric form were responsible for the binding. Another explanation involves alternative binding of the dimer. One binding theory involves cooperative binding of two monomeric insulin molecules thus suggesting that two insulin molecules bind at the same time [50], [52]. This theory involves two binding sites, the classical binding site and the second binding site. The classical binding site mainly consist of positions in the C-terminal end of the B-chain and hydrophobic residues located in the B-chain's α-helix together with the C-terminal end of the A-chain buried beneath the C-terminus of the B-chain. The second binding site with lower binding affinity [50], consisting of A13L and B17L, is located in the hexamer forming surface. In the B25C-dimer the classical binding site is not accessible to binding; both because of the overlap between the dimer forming surface and the binding surface, but also because it is believed that binding of insulin to the receptor involves a shift in the C-terminal end of the B-chain exposing the hydrophobic residues involved in binding. This is not possible in the dimer form [53]–[56]. Obstruction of the classical binding site by removal of the first four amino acids in the A-chain (desA1-4) was shown to decrease binding affinities to 0.014% relative to HI [57] and fixation of the C-terminal end of the B-chain in SCI resulted in a loss of *in vivo* activity [55]. In contrast, the second binding site is still fully exposed and available for receptor binding. To explain B25C-dimer receptor affinity and lipogenesis results by binding exclusively to the second binding site would necessitate that binding solely to this binding site leads to receptor activation, and this has still not been shown. Regardless of which explanation for the B25C-dimer insulin receptor binding is correct, it is clear that insulin dimer is not able to bind to the insulin receptor with the same affinity as the insulin monomer. This supports the hypothesis that it is the monomeric form of insulin that is responsible for high affinity interaction with insulin receptor.

The stability of the B25C-dimer became evident during conversion of the expressed precursor to the mature insulin analogue. The compact structure achieved during dimer formation likely prevents proteases from accessing the cleavage site. The C-terminal of the B-chain is known to be prone to protease degradation [58]. It is also the only part of insulin known to form β-strand structure. Comprehensive analysis of binding motives recognised by proteases based on >1500 3D structures showed that proteases with a few exceptions recognize extended β-strand conformations [59]. B25C-dimer hinders the access to the β-strand structure so the enhanced stability against ALP will most likely also reflect enhanced stability against other proteases.

The stability against fibrillation was also markedly improved for the B25C-dimer compared to HI. It has been suggested that hexamer formation during storage in secretory granules protects insulin against fibrillation *in vivo* [48] and this is often exploited when formulating insulin analogues for clinical use by addition of zinc [10]. The ThT assay results with the B25C-dimer supported that fibrillation only occurs through the monomeric form as no fibrillation was seen for the dimer. Thus, it is clear that a stable dimeric form is sufficient to stabilize insulin against fibrillation and denaturation. This is also supported by the higher stability of the B25C-dimer compared to HI hexamer as seen in the DSC results. Thus, it is possible to generate an insulin dimer which is sufficiently stable against denaturation and fibrillation under stressed conditions such as high temperature making the hexameric form redundant in protection against denaturation and fibrillation. Stabilizing the insulin dimer formation, will however result in limited receptor binding affinity. The evolutionary solution to this fine balance between stability and receptor binding is the formation of insulin hexamers. In the insulin hexamer, the relatively weak insulin dimer interaction is stabilized by zinc-induced association of three insulin dimers while allowing for sufficient concentration of insulin monomer at low concentrations without zinc and thus ensuring high affinity receptor binding. This system has been preserved through evolution of most vertebrate species. The exception to this rule is found in guinea pigs insulin that has a mutation in the B10 position from a histidine residue to an aspartate residue and is therefore not capable of forming zinc induced hexamers. Its inability to form dimers was also shown by AUC [60]. It is believed that removal of the selective constraints associated with the ability to oligomerize has allowed for additional mutations to compensate for the loss in stability associated with loss in oligomerization. The monomeric form of guinea pig insulin is stabilized by replacement of hydrophobic residues on the surface (involved in oligomerization) with hydrophilic residues [61]. These introduced mutations, however, lead to reduced potency [62], which is compensated by significantly increased level of insulin receptors in guinea pigs [63].

In this paper, we describe a novel covalent insulin dimer that is structurally identical to the self-association dimer of human insulin and further associates into a hexamer in the presence of zinc similarly to HI. We used this covalent dimer to directly investigate the structure-function relationships of insulin oligomerization to stability and receptor binding. This covalent dimer did not bind to the insulin receptor. However, significant improvements in stability of B25C-dimer relative to HI were demonstrated by markedly slower processing of the precursor with ALP, an almost 40°C increase in T_m_, more than 44 kJ/mol increase in ΔH in DSC measurements and no fibrillation in ThT assay. Our results underline that the dimerization of insulin is responsible for insulin stability, while the monomeric form is required for this hormone's activity.

## Supporting Information Legends

Figure S1 **Positions in the dimer forming surface with Cα to Cα distance <10 Å.** The two B-chains in the dimer from PDB file 1MSO are shown in grey with the positions B12(purple), B13(cyan), B24(blue), B25(pink), B26(yellow) shown with the respective distances between the position in each of the B-chains.
(TIF)

Figure S2 **The UV chromatogram (215 nm) of partially purified B25C precursor.** The LC/MS analyses of the pool after partial purification of B25C precursors using cation exchange chromatography showed that two precursors, peak 1 and peak 2, were found after expression. The masses for both peaks corresponded to a B25C-dimer linked by a disulfide bond.
(TIF)

Figure S3 **Pulse chase experiment of the B25C-dimer.** The covalently linked B25C-dimer is seen as a strong band between the 14.3 kDa and 21.5 kDa marker. Unprocessed precursor containing the leader is seen for the dimer as a band at the 30 kDa marker for the samples treated with PNGase.
(TIF)

Figure S4 **Characterization of by-product.** **A:** Total ion count chromatogram of reduced by-product. **B:** MS/MS in source fragmentation spectrum of peak with a mass equal to containing single chain insulin (A-chain+B-chain-H_2_O(18 Da)). The b-ions were identified from all fragments in black. The fragments in gray were identified by either an a-ion in the N-terminal or y-ions in the C-terminal. The MS/MS analyses showed the by-product to be a result of ALP's transpeptidase activity, where a peptide bond between B29 and A1 was formed. ALP is known not only to work as a protease but also as a transpeptidase [64]. Linking of the B-chain's C-terminal to the A-chain's N-terminal by a peptide bond was also observed before using trypsin another transpeptidase [55].
(TIF)

Figure S5 **Sedimentation Velocity experiments illustrating the self-association abilities of the B25C-dimer compared to HI.** **A:** In the absence of zinc ions. **B:** In the presence of increasing amount of zinc ions. The B25C-dimer had a typical pattern for a reversible self-association, qualitatively similar to HI both in the absence and presence of zinc ions.
(TIF)
