# Supplementary figures and images for: Novel Covalently Linked Insulin Dimer Engineered to Investigate the Function of Insulin Dimerization
Source: PLoS One. 2012 Feb 17;7(2):e30882. doi: 10.1371/journal.pone.0030882 (PMC3281904; doi:10.1371/journal.pone.0030882)

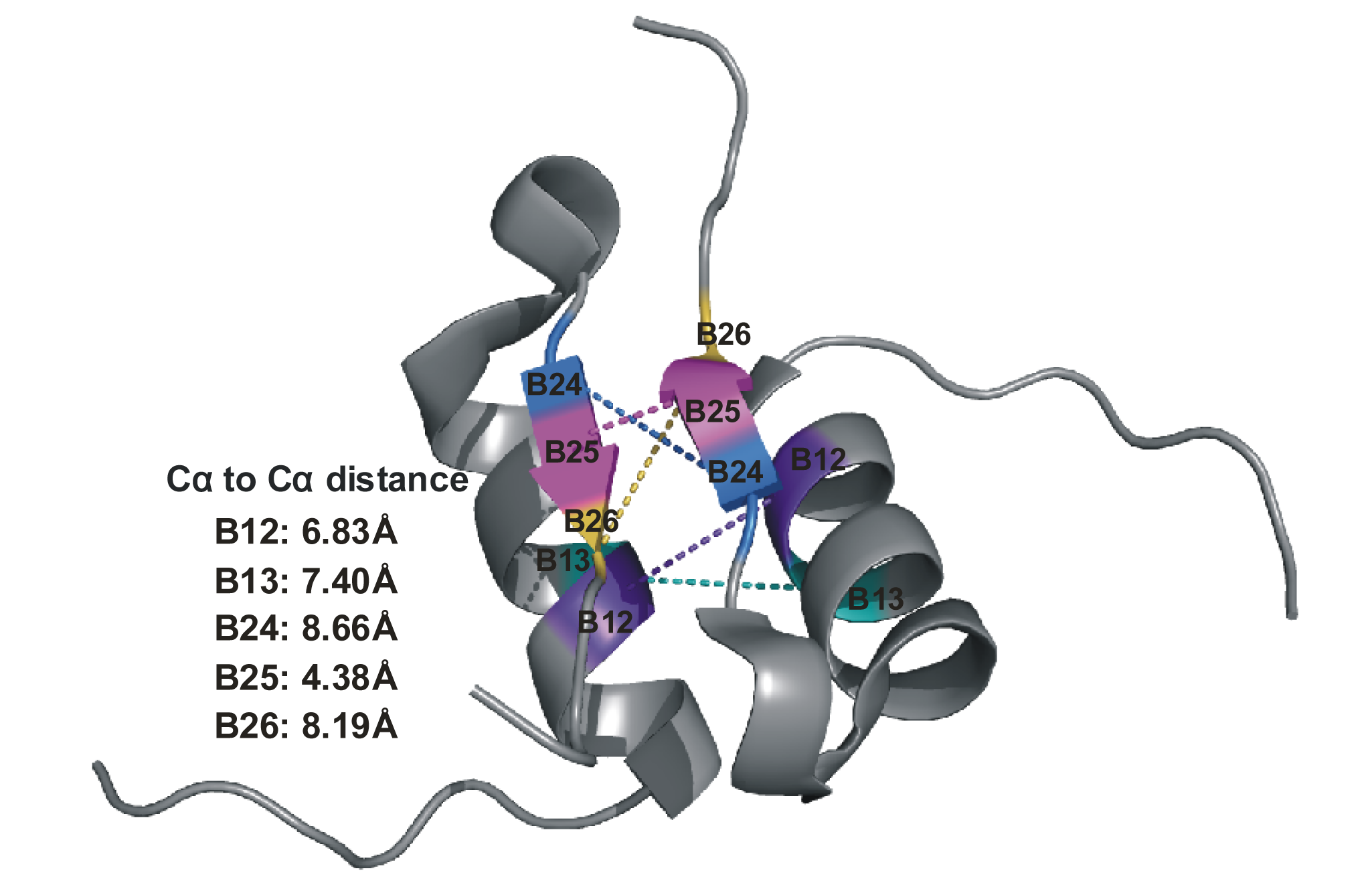

Supplement: Figure S1 — Positions in the dimer forming surface with Cα to Cα distance <10 Å. The two B-chains in the dimer from PDB file 1MSO are shown in grey with the positions B12(purple), B13(cyan), B24(blue), B25(pink), B26(yellow) shown with the respective distances between the position in each of the B-chains. (TIF) [file pone.0030882.s001.tif]

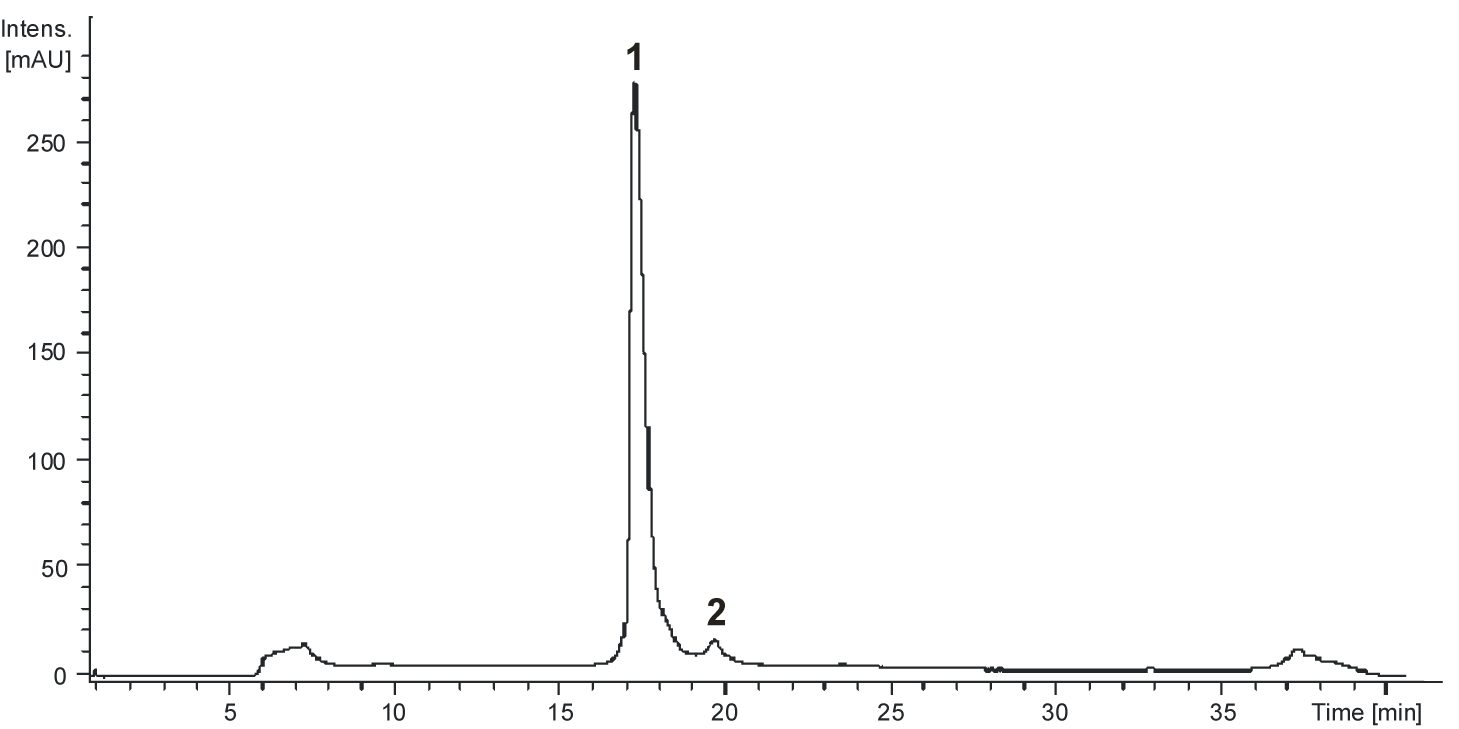

Supplement: Figure S2 — The UV chromatogram (215 nm) of partially purified B25C precursor. The LC/MS analyses of the pool after partial purification of B25C precursors using cation exchange chromatography showed that two precursors, peak 1 and peak 2, were found after expression. The masses for both peaks corresponded to a B25C-dimer linked by a disulfide bond. (TIF) [file pone.0030882.s002.tif]

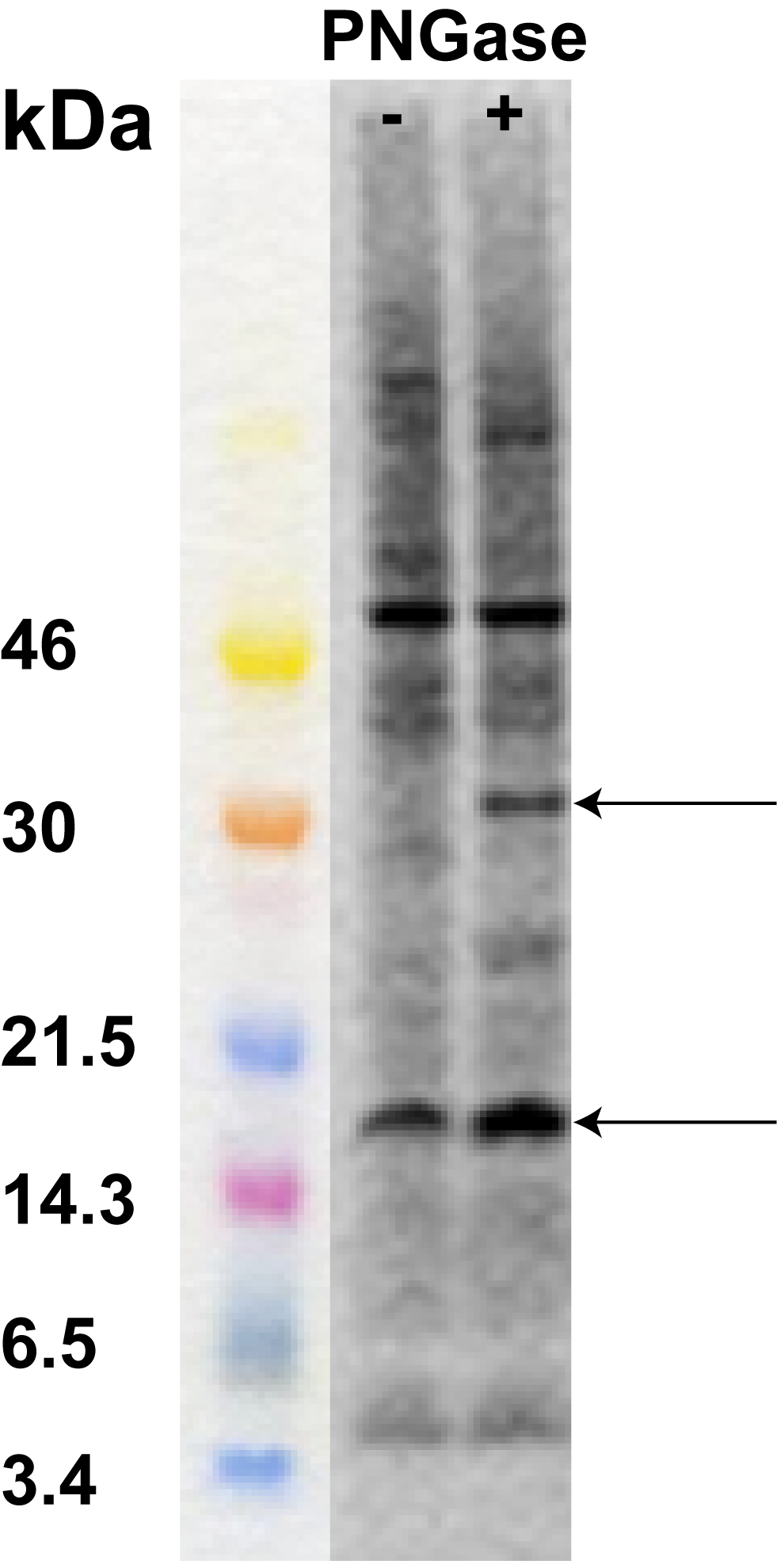

Supplement: Figure S3 — Pulse chase experiment of the B25C-dimer. The covalently linked B25C-dimer is seen as a strong band between the 14.3 kDa and 21.5 kDa marker. Unprocessed precursor containing the leader is seen for the dimer as a band at the 30 kDa marker for the samples treated with PNGase. (TIF) [file pone.0030882.s003.tif]

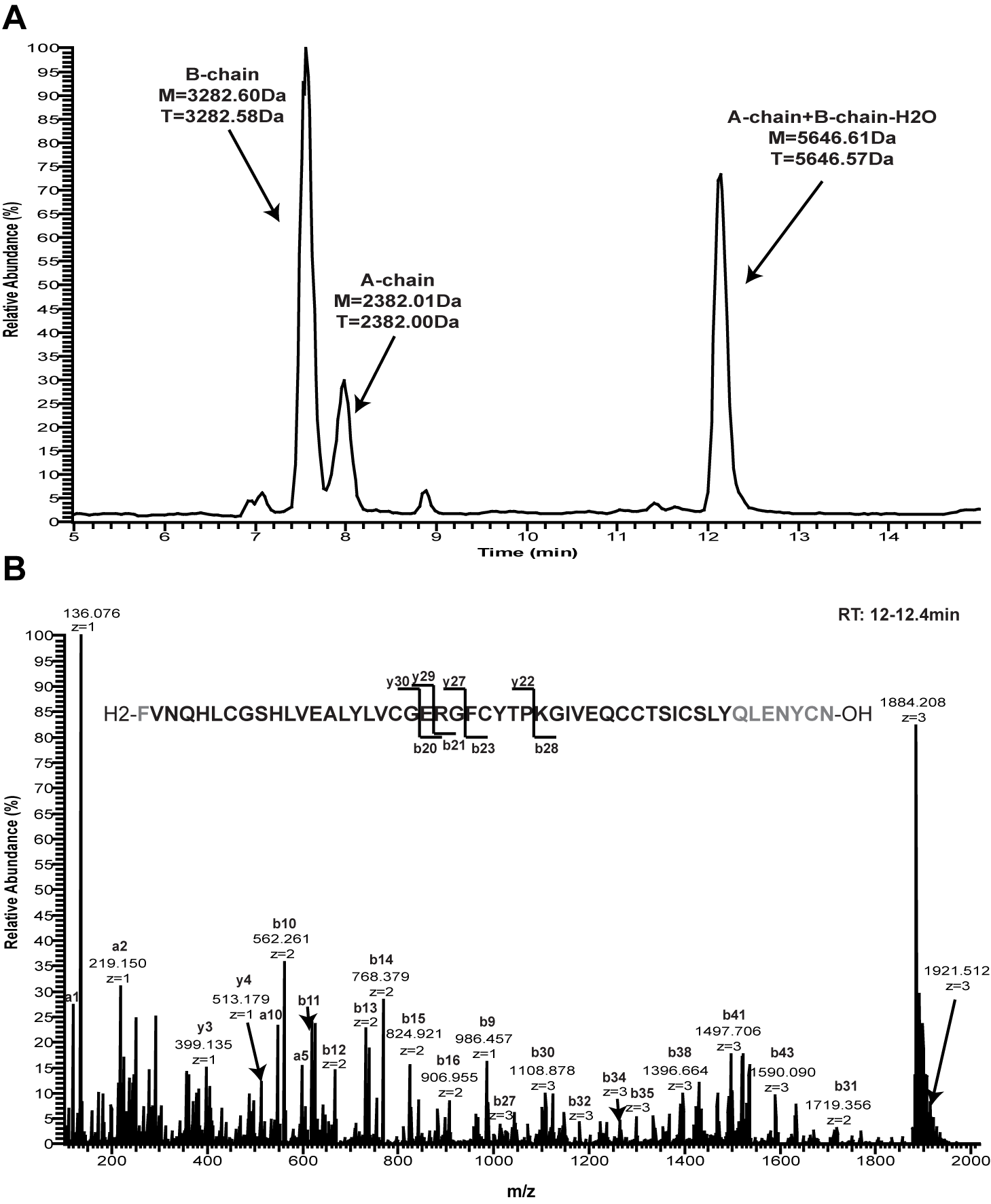

Supplement: Figure S4 — Characterization of by-product. A: Total ion count chromatogram of reduced by-product. B: MS/MS in source fragmentation spectrum of peak with a mass equal to containing single chain insulin (A-chain+B-chain-H2O(18 Da)). The b-ions were identified from all fragments in black. The fragments in gray were identified by either an a-ion in the N-terminal or y-ions in the C-terminal. The MS/MS analyses showed the by-product to be a result of ALP's transpeptidase activity, where a peptide bond between B29 and A1 was formed. ALP is known not only to work as a protease but also as a transpeptidase [64]. Linking of the B-chain's C-terminal to the A-chain's N-terminal by a peptide bond was also observed before using trypsin another transpeptidase [55]. (TIF) [file pone.0030882.s004.tif]

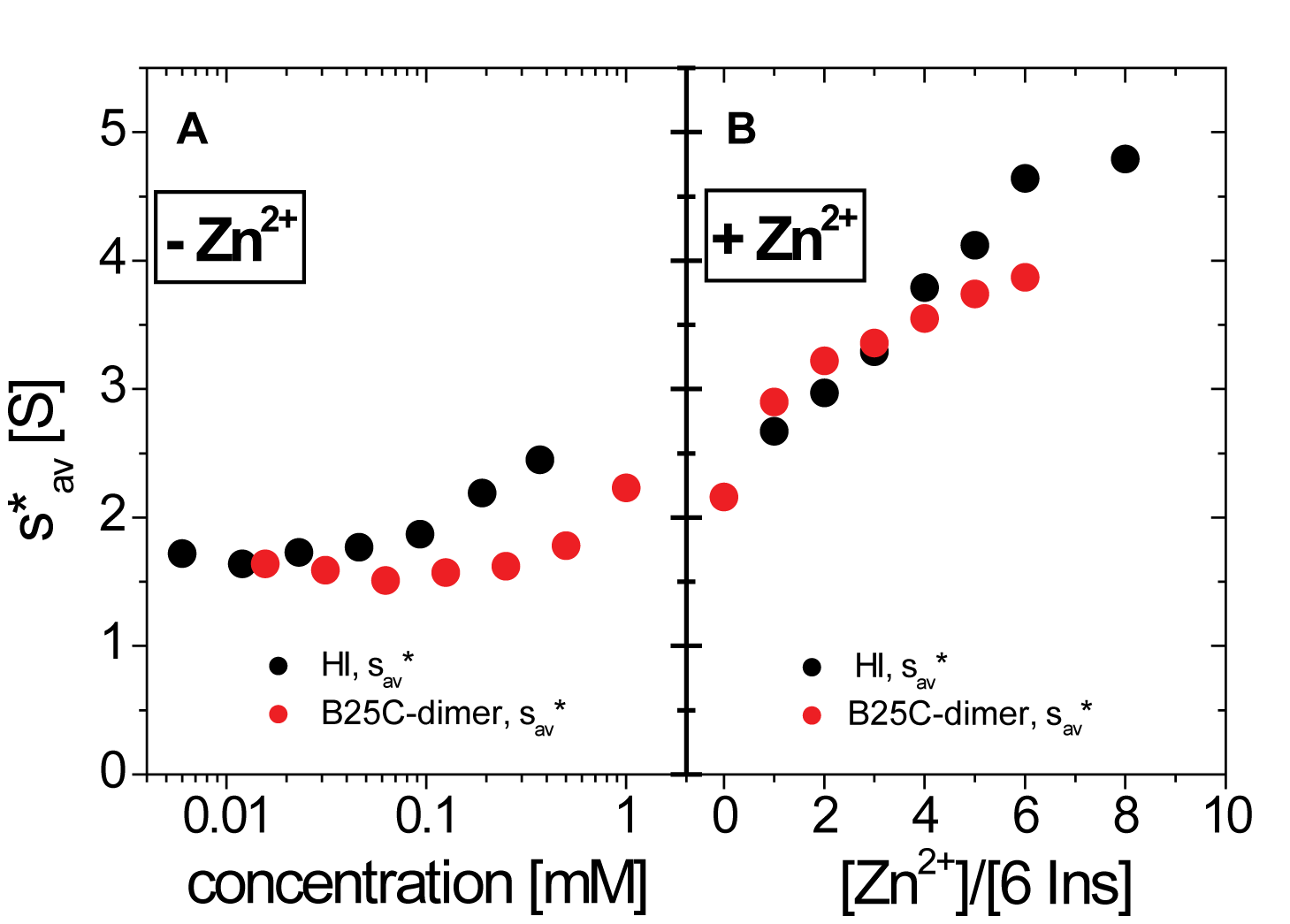

Supplement: Figure S5 — Sedimentation Velocity experiments illustrating the self-association abilities of the B25C-dimer compared to HI. A: In the absence of zinc ions. B: In the presence of increasing amount of zinc ions. The B25C-dimer had a typical pattern for a reversible self-association, qualitatively similar to HI both in the absence and presence of zinc ions. (TIF) [file pone.0030882.s005.tif]
